# How synergy between mechanistic and statistical models is impacting research in atrial fibrillation

**DOI:** 10.3389/fphys.2022.957604

**Published:** 2022-08-30

**Authors:** Jieyun Bai, Yaosheng Lu, Huijin Wang, Jichao Zhao

**Affiliations:** ^1^ Guangdong Provincial Key Laboratory of Traditional Chinese Medicine Information Technology, Jinan University, Guangzhou, China; ^2^ College of Information Science and Technology, Jinan University, Guangzhou, China; ^3^ Auckland Bioengineering Institute, University of Auckland, Auckland, New Zealand

**Keywords:** digital twin, atrial fibrillation, heart rhythm, computational modelling, artificial intelligence, machine learning, catheter ablation, anti-arrhythmic drugs

## Abstract

Atrial fibrillation (AF) with multiple complications, high morbidity and mortality, and low cure rates, has become a global public health problem. Although significant progress has been made in the treatment methods represented by anti-AF drugs and radiofrequency ablation, the therapeutic effect is not as good as expected. The reason is mainly because of our lack of understanding of AF mechanisms. This field has benefited from mechanistic and (or) statistical methodologies. Recent renewed interest in digital twin techniques by synergizing between mechanistic and statistical models has opened new frontiers in AF analysis. In the review, we briefly present findings that gave rise to the AF pathophysiology and current therapeutic modalities. We then summarize the achievements of digital twin technologies in three aspects: understanding AF mechanisms, screening anti-AF drugs and optimizing ablation strategies. Finally, we discuss the challenges that hinder the clinical application of the digital twin heart. With the rapid progress in data reuse and sharing, we expect their application to realize the transition from AF description to response prediction.

## 1 Introduction

The most common sustained arrhythmia atrial fibrillation (AF) not only has high morbidity and mortality, but also is very difficult to prevent, diagnose and treat, bringing a huge economic burden to individuals, countries and society (Hindricks et al., 2020). AF is often asymptomatic and frequently undetected clinically (Gibbs et al., 2021), but it increases the risk of stroke by fivefold (Freedman et al., 2016), heart failure by threefold (Kotecha and Piccini, 2015), and mortality by twofold (Tsao et al., 2022). Occurring in less than 0.16% in patients aged≤ 49 years, AF has a prevalence that increases steadily with advancing age, affecting up to 9% in those aged ≥65 years and 17% in patients beyond the age of 80 years (Freedman et al., 2021); the overall lifetime risk is at least 37.8% (Staerk et al., 2018). The number of individuals affected by AF had exceeded 46.3 million in 2016, with more than five million each year new cases diagnosed, as well as the number will double by 2060 (Krijthe et al., 2013). The costs associated with AF are large: in the U.S. alone, the incremental cost of AF treatment exceeds $26.0 billion (Kim et al., 2011), while the incremental cost of asymptomatic AF exceeds $3.1 billion (Turakhia et al., 2015). Thus, AF has become a global public health problem.

The 2020 European Society of Cardiology guidelines endorse the Atrial Fibrillation Better Care (ABC) pathway as a structured approach for AF management, addressing three principal elements: “A” - avoid stroke (with oral anticoagulation), “B” - patient-focused better symptom management, and “C” - cardiovascular and comorbidity risk factor reduction and management (Hindricks et al., 2021). The mobile AF application randomized trial confirmed that the ABC approach could reduce adverse outcomes more significantly than usual care (Guo et al., 2020). In addition, several studies found that implementing the ABC pathway can improve cure rates, decrease related costs and the risk of complications, and reduce mortality and morbidity (Pastori et al., 2019; Yoon et al., 2019; Wijtvliet et al., 2020). Despite significant advances in the management and treatment of AF using the ABC pathway, AF continues to pose a significant risk of death, partly due to knowledge gaps in the fundamental AF mechanisms and treatment strategies (Goette et al., 2019). Developing a personalized digital twin of the heart, which integrates coherently and dynamically the patient’s clinical data over time, will likely be essential to overcome current challenges (Corral-Acero et al., 2020; Lindemans, 2020; Gerach et al., 2021). Over the last decades, the digital twin heart has emerged as a modality to diagnose, understand and therapy complex arrhythmias (Gillette et al., 2021a; Gillette et al., 2021b). This mini-review is structured as follows: Section 2 briefly summarizes the AF pathophysiology and current therapeutic modalities. Section 3 summarizes the achievements of synergy between mechanistic and statistical models in three aspects: understanding AF mechanisms, screening anti-AF drugs and optimizing ablation strategies. Finally, we discuss the challenges that hinder the clinical application of synergy between mechanistic and statistical models. More methodological details on mechanistic and (or) statistical models can refer to other reviews (Nattel et al., 2021a; Heijman et al., 2021b; Nattel et al., 2021b; Leblanc et al., 2021; Trayanova et al., 2021).

## 2 Atrial fibrillation pathophysiology and current therapeutic landscape

**FIGURE 1:**
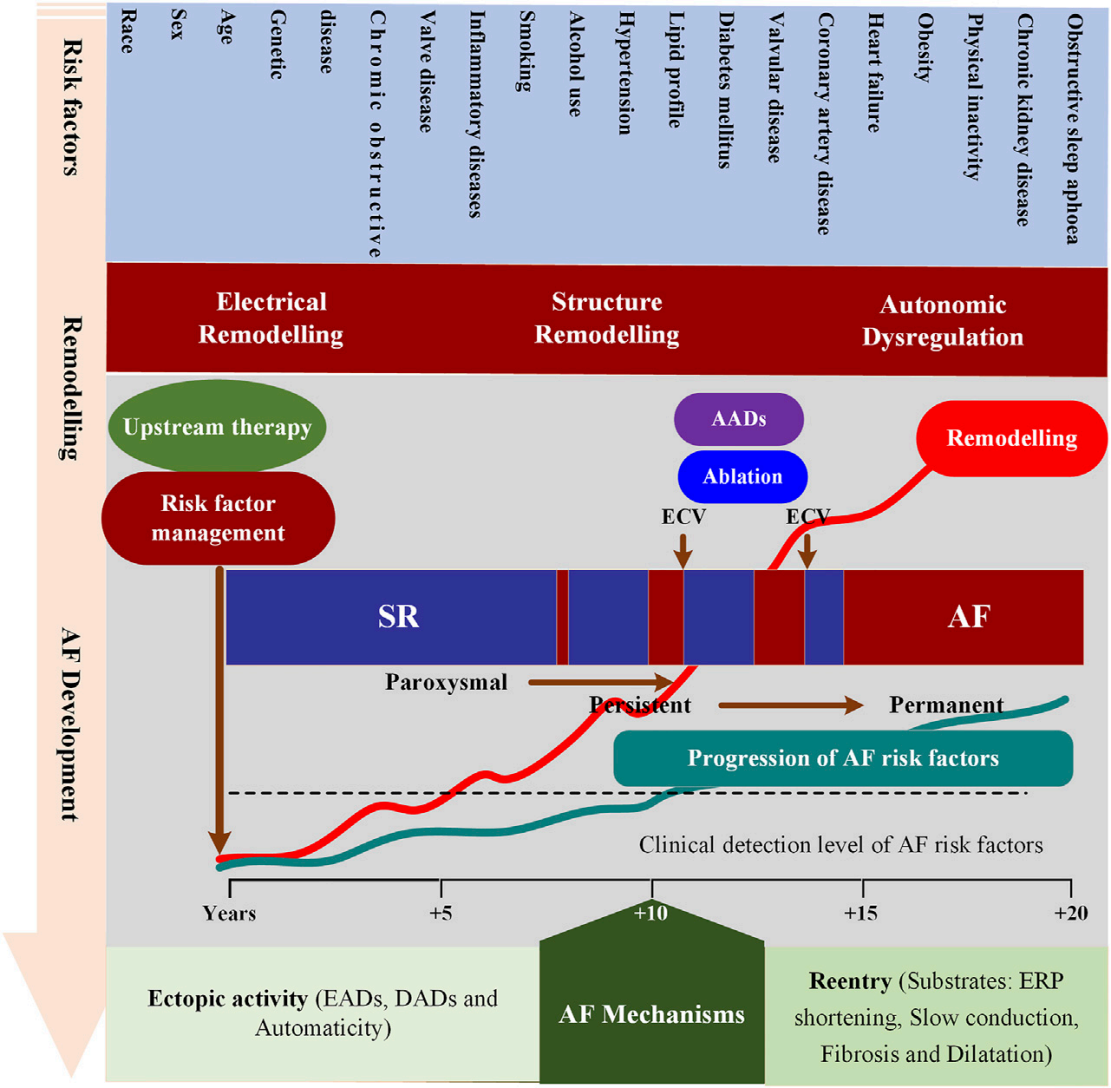
Schematic overview of mechanisms underlying AF development and progression. This figure depicts the interrelationships between risk factors, time-dependent atrial remodeling and progression from sinus rhythm (SR) through paroxysmal and persistent to permanent AF. ECV = electrical cardioversion; ERP = effective refractory period; AADs = antiarrhythmic drugs; EADs = Early afterdepolarization; DADs = Delayed afterdepolarization.

Many dynamic predisposing factors, including modifiable and non-modifiable risk factors, contribute to the onset and progression of AF. The identified non-modifiable risk factors include age, sex, ethnicity and genetics, while modifiable factors consist of smoking, alcohol consumption, hypertension, lipid profile, diabetes, vascular disease, coronary artery disease, heart failure, obesity, physical inactivity, chronic kidney disease, obstructive sleep apnoea, chronic obstructive pulmonary disease, valve disease and inflammatory diseases (Benjamin et al., 1994; Mont et al., 2008; Lau et al., 2017; Roselli et al., 2020). These risk factors can lead to atrial remodeling through various pathways facilitating the development of AF. The atrial remodeling can be grouped into electrical, structural, and autonomic remodeling that allows for the initiation and maintenance of AF. Recent reviews detailing the role of each risk factor in the pathophysiology of AF and various underlying mechanisms can be summarized as follows (Dobrev et al., 2019; Nattel et al., 2020): Complex electrical defects in the atria, including reentrant waves and localized premature atrial contractions, contribute to the development of AF. Among them, premature atrial beats are mainly derived from the early and late afterdepolarization (EAD/DAD) of atrial cells, and reentrant waves are related to the shortening of the effective refractory period, slow conduction and conduction barriers (Hansen et al., 2015; Mikhailov et al., 2021). AF is not only a complex multifactorial disease, but also a progressive condition, moving from paroxysmal AF (self-terminating in <7 days), persistent AF (lasting >7 days and requiring termination by cardioversion) to long-standing persistent AF (lasting >1 year and requiring a rhythm control strategy) and, may become resistant to antiarrhythmic drugs (AADs) (Chiang et al., 2012) and ablation therapies (Wyse et al., 2014; Ogawa et al., 2018). In addition to advancing age and the progressive remodeling caused by modifiable risk factors (Mountantonakis et al., 2012), AF progression also has a substantial genetic component (e.g., the most common ones at 4q25 near PITX2) (Gudbjartsson et al., 2007) (Figure 1). However, the contribution of each factor in a specific patient to AF occurring and progression remains incompletely understood.

Potential AF patients are usually diagnosed with long-term electrocardiogram (ECG) monitoring to determine the temporal patterns (Hindricks et al., 2020). In addition to AF patients with distinct ECG features, up to 40% of AF patients have no obvious symptoms (Page et al., 2003; Jones et al., 2020). A large number of undiagnosed AF patients cannot receive the necessary risk management (Davidson et al., 2022), resulting in irreversible AF-causing structural remodeling, increasing the difficulty of later treatment and reducing therapeutic efficacy. The EAST-AFNET4 trial has confirmed that early rhythm-control therapy can reduce the risk of adverse outcomes (Kirchhof et al., 2020). Although AF screening is also recommended, the best way to screen is uncertain.

Rate and rhythm control strategies are two cornerstones of symptomatic AF management. For preventing mortality and morbidity from cardiovascular causes, the effectiveness of the two strategies is comparable (Van Gelder et al., 2002; Wyse et al., 2002). Due to the limited efficacy and proarrhythmic side effects. AADs are widely used but cannot effectively control sinus rhythm (Heijman et al., 2021a). Although pulmonary vein isolation (PVI) via catheter ablation (CA) can improve sinus rhythm maintenance compared to AADs (Marrouche et al., 2018; Kelly et al., 2019), many AF recurrence cases illustrate that the one-size-fits-all approach is still suboptimal (Andrade et al., 2019). These studies found patients with later AF recurrences respond better to AADs and repeat ablation, providing metrics to assess different CA strategies (i.e., the time to AF recurrence) (Gaztañaga et al., 2013). Despite the increasing importance of CA strategies (Asad et al., 2019; Blomström-Lundqvist et al., 2019), AADs remain an important component of AF management (Markman et al., 2020; Andrade et al., 2021), since a large number of AF patients, and the costs and risks of the invasive procedures of CA should be considered. However, the choice of AADs is limited by their proarrhythmic and toxic properties (Zimetbaum, 2012). Therefore, specific rate or rhythm control strategies for distinct fundamental molecular and cellular determinants of AF are likely to yield better therapeutic outcomes (Garvanski et al., 2019). Nevertheless, it is challenging to predict which AF patients are likely to recur and thereby require more aggressive therapy.

## 3 Applications of digital twin techniques in atrial fibrillation management

Digital twin technologies are expected to overcome existing difficulties. The digital twin was firstly presented by Michael Grieves in 2003 and was initially described as a virtual representation of a physical product (Grieves, 2005). Its definition was expanded to consist of three components: a physical product, its virtual representation and a two-way data connection between the virtual and the physical representations (Haag and Anderl, 2018). The digital twin in health care denotes the vision of “a comprehensive, virtual tool that integrates coherently and dynamically the clinical data acquired over time for an individual using statistical models and mechanistic modeling and simulation” (Alber et al., 2019). Using digital twin techniques, precision cardiology will be provided in a collaborative way, through mechanistic modeling and simulation of multiscale heart and the use of statistical models learned from massive raw data (including simulated, experimental and clinical data) (Bai et al., 2016; Bai et al., 2017a; Bai et al., 2017b). Following fundamental biophysical laws and concepts, mechanistic models integrate fragmented data into a “biologically functional heart” that can be used to simulate cardiac electrophysiological dynamics to explore underlying mechanisms (Bai et al., 2021a). However, it is a difficult task to reduce hundreds of thousands of multiscale simulation data to meaningful predictive biomarkers, and clinical biomarkers or quantitative measures of structural remodeling derived from raw imaging data were not considered in mechanistic modeling. Statistical models are ideal for identifying meaningful predictive biomarkers in high-dimensional simulation and clinical data (Corral-Acero et al., 2020; Liu et al., 2021; Zeng et al., 2021; Zhong et al., 2022). Therefore, digital twin techniques have value in evidence generation, diagnosis and treatment.

Although personalized atrial computer models from either imaging data or electroanatomical maps have been developed, their standardization has just begun. Lately, Razeghi et al. published the CemrgApp platform for image processing to provide MRI segmentation, including fibrotic tissue distribution derived from late gadolinium enhancement (LGE) intensity in a semi-automatic and userfriendly way (Razeghi et al., 2020). In addition, Williams et al. presented the OpenEP framework for evaluating electroanatomic mapping data (Williams et al., 2021). Considering the advantages of CemrgApp and OpenEP, Azzolin et al. proposed a patient-specific Augmented Atria generation pipeline (AugmentA) that ingests the tomographic segmentations and (or) the electroanatomic map, and provided ready-to-use atrial personalized computational models from clinical data. AugmentA consists of a preprocessing step (Azzolin et al., 2021a), atrial orifices’ annotation, a statistical shape model fitting procedure, fiber generation (Zheng et al., 2021) and conduction velocity (CV) estimation. AugmentA offers an automated and comprehensive pipeline delivering personalized atrial computer models from clinical data in procedural time (Azzolin et al., 2022a). This is a step forward toward standardized assessment of arrhythmia vulnerability and testing of ablation strategies. The following part of the review addressed studies using digital twin techniques for understanding AF mechanisms, screening anti-AF drugs and optimizing AF ablation strategies.

### 3.1 Understanding AF mechanisms using digital twin techniques

Recently, several hybrid studies utilizing both mechanistic and statistical approaches investigated AF mechanisms. An example of the use of digital twin techniques is investigations of atrial electrophysiological variability (Muszkiewicz et al., 2016). Although the variability is manifested through functional differences between individuals and has important implications for AF progression, it is often ignored in traditional studies by averaging samples from multiple individuals (Bai et al., 2018). Recently, a digital twin framework has been designed to study its underlying mechanisms and arrhythmogenic risks under different conditions (Ni et al., 2020). Based on the common assumption of heterogeneous current properties and an appropriate atrial cell model, parameters of the baseline model are varied to construct a population of candidate models by using different sampling methods (e.g., Latin Hypercube sampling (Burrage et al., 2015), sequential Monte Carlo (Lawson et al., 2018) and Bayesian history matching (Coveney and Clayton, 2018)). Populations of models (POMs) are directly calibrated to experimental data distributions to provide valuable tools for investigating the factors that underlie emergent atrial electrophysiology. In detail, experimentally-calibrated POMs are used to conduct simulations of atrial electrophysiology, whereas statistical models are used to identify how variability in in-silico atrial electrophysiology modulates the dynamics of AF.

At the cellular level, several studies concentrated on identifying potential determinants of inter-subject variability in calcium transient (Muszkiewicz et al., 2018; Vagos et al., 2021), action potential (AP) duration (APD) (Sánchez et al., 2014; Chang et al., 2017; Coveney and Clayton, 2020; Nesterova et al., 2020), triggered activity (Morotti and Grandi, 2017; Zhu et al., 2021) and dynamic AP restitution (Vagos et al., 2017). In these studies, the kinetic parameters influencing ion currents (Chang et al., 2017) and ionic conductances (Sánchez et al., 2014; Coveney and Clayton, 2020; Nesterova et al., 2020) were identified to have a strong influence on APD and Dome potential. In addition to ionic current properties, external factors (e.g., stimulus strength) were also found to modulate AP amplitude and APD (Muszkiewicz et al., 2014). Digital twin techniques were also used to classify different AF types, such as AFs at different ages (Nesterova et al., 2020), as well as upregulated vs downregulated Pitx2-induced AFs (Zhu et al., 2021). At the tissue level, factors related to the maintenance and formation of reentrant waves were investigated. For example, the study of Simon et al. employed a population of tissue models to identify inter-subject variability that modulates CV that is critical for arrhythmia inducibility (Simon et al., 2017), while the study of Clayton et al. investigated the influence of the spatial scale of fibrosis regions on the APD dispersion and vulnerability to re-entry (Clayton, 2018). They found that the specific balance between sodium current and diffusion coefficient can promote the formation of reentrant waves, and small fibrosis areas favor the maintenance of reentrant waves. The potential of the digital twin heart in exploring AF mechanisms was directly highlighted in these studies reviewed in this section.

### 3.2 Screening anti-AF drug using the digital twin techniques

A variety of computational models have been used to screen anti-AF drugs. Some of them are related to potential drug targets, as is the case of Liberos et al., who used chronic AF-induced remodeling tissue models to investigate the effect of each remodeled target on rotor dynamics. The study found that the effectiveness of *I*_*CaL*_ block as a rhythm control strategy depends on the availability of *Na* ^*+*^ and *Ca* ^*2+*^ currents (Liberos et al., 2016; Liberos et al., 2017). In addition, special ion channels as drug targets (including *I*_*Na*_ and/or *I*_*NaL*_, *I*_*Kr*_, *I*_*Kur*_, *I*_*K,Ach*_, *I*_*K,2P*_ and *I*_*K,Ca*_) were investigated by altering the conductance or the gating kinetics. Scholz et al. introduced a mathematical description of *I*_*Kur*_ blockade into models of normal and remodeled atrial electrophysiology and found antiarrhythmic effects of *I*_*Kur*_ inhibitors are dependent on kinetic properties of blockade (Scholz et al., 2013). Schmidt et al. changed the conductance of *I*_*K,2P*_ to investigate the effects of genetic ablation of TASK-1 and found antiarrhythmic effects of anti-TASK-1-siRNA were associated with APD prolongation (Schmidt et al., 2019). Using a population of virtual whole-atria human models, Sánchez et al. found specific inhibitions of *I*_*K1*_ *, I*_*NaK*_ *,* or *I*_*Na*_ may be a promising rhythm control strategy by enlarging wave meandering to reduce the dominant frequency (Sánchez et al., 2017). Another interesting study by Ni et al. investigated the synergistic anti-AF effects of the combined block of multiple atrial-predominant *K* ^*+*^ currents using populations of cell and tissue models. The study found that the proposed strategy can promote favorable positive rate-dependent APD prolongation, illustrating its potential anti-AF effects (Ni et al., 2020). Some other studies concentrated on predicting the risk of anti-AF drugs. In the study by Bai et al., the focus was on evaluating the efficacy of disopyramide, quinidine, and propafenone on Pitx2-induced AF. The study found that disopyramide is most effective in the three drugs for Pitx2-induced AF by prolonging the wavelength (Bai et al., 2021b). Wiedmann et al. tested the antiarrhythmic effects of the high-affinity TASK-1 inhibitor A293 on cardioversion in a porcine model of paroxysmal AF and multicellular tissue modeling predicted that the antiarrhythmic effect of TASK-1 inhibition by A293 was strongly dependent on the tissue conductivity and the resulting CV (Wiedmann et al., 2020). Loewe et al. evaluated the dynamic effects of amiodarone and dronedarone on human atrial patho-electrophysiology and simulated results provided possible explanations for the superior efficacy of amiodarone (Loewe et al., 2014). The digital twin techniques also were used to classify drugs. For example, Sanchez et al. predicted the effects of isoproterenol, flecainide and verapamil using in silico simulations and then classified these drugs based on proarrhythmic patterns using a random forest algorithm. The study found that *I*_*K1*_ is the most important current for classifying the proarrhythmicity of a given profile (Sanchez de la Nava et al., 2021). These initial results point to future developments where the combination of mechanistic and statistical models could create efficient platforms for drug screening and cardiotoxicity studies, and, importantly, platforms for individualized medication.

### 3.3 Optimizing AF ablation strategies using the digital twin techniques

Pulmonary vein isolation (PVI) by cardiac ablation emerged as a feasible strategy in AF ablation and has evolved from segmental ostial pulmonary vein ablation to the guide ablation with the 3D electroanatomical mapping, to wide-area circumferential ablation with verification of conduction block. For long-term ablation success, PVI using point-by-point radiofrequency or with the cryoballoon has evolved substantially, with multiple energy sources and a variety of ablation tools being available to make it safe and effective. These emerging tools include numerous novel radiofrequency catheters (such as Satake HotBalloon, Heliostar, Luminize-RF, Sphere-9 catheter and NADH autofluorescence-guided ablation catheter) and alternative energy sources (e.g., endoscopic laser balloon and pulsed field electroporation). Although PVI has been shown to have a high success rate in patients with paroxysmal AF in proximity to the PV regions, it is insufficient in the most patients with persistent AF outside the PV regions. Over the past 2 decades, numerous anatomical structures have been suggested as sites from which non-pulmonary vein triggers might occur, including the posterior wall of the left atrium, the left atrial appendage, the superior vena cava, the crista terminalis, the fossa ovalis, the coronary sinus, the ligament of Marshall and adjacent to the atrioventricular valve annuli. Unfortunately, strong evidence to support improved clinical outcomes for any adjunctive ablation strategies is lacking and identifying functional localized target sites for ablation remains challenging (Wu et al., 2021). This may be optimized by using digital twin techniques.

One of the applications of digital twin techniques is to link biomarkers to tissue properties. For example, Corrado et al. found combing CV and APD with the atrial surface area can improve the accuracy in identifying regions that tether re-entrant activation patterns using both biophysically detailed computational models of the atria and a support vector machine classifier (Corrado et al., 2021). Godoy et al. linked body surface potential mapping (BSPM) derived indexes to the location of ectopic foci, indicating its potential application of these biomarkers in targeting ectopic foci (Ferrer-Albero et al., 2017; Godoy et al., 2018a; Godoy et al., 2018b).

Another application is to identify potential ablation targets. In these studies, mechanistic models were used to simulate the typical AF scenarios and statistical models were used to find the regions in the atria where arrhythmias are inducible (Sha et al., 2022). For example, Ravikumar et al. evaluated the performance of multiscale frequency [MSF], Shannon entropy [SE], kurtosis [Kt], and multiscale entropy [MSE] techniques to identify the pivot point of the rotor using unipolar and bipolar EGMs obtained from numerical simulations (Ravikumar et al., 2021). Ganesan et al. developed and evaluated the AF source area probability (ASAP) mapping algorithm in 2D and 3D atrial simulated tissues with various arrhythmia scenarios and a retrospective study with three cases of clinical human AF. They found that ASAP delineated the AF source in over 95% of the simulated human AF cases within less than eight catheter placements regardless of the initial catheter placement (Ganesan et al., 2020). The study of Sánchez et al. characterized atrial fibrotic substrate with a hybrid in silico and *in vivo* dataset and found the digital twin techniques can overcome a single voltage cut-off value to identify fibrotic tissue from intracardiac signals (Sánchez et al., 2021). Using personalized biophysically detailed computational models of the atria based on the patient’s LGE-MRI, Zahid et al. employed mechine learning to determine the characteristics of fibrosis distribution and found the ablation targets may be the regions with high fibrosis density and entropy (Zahid et al., 2016). And this approach has been shown to be more accurate than these purely image-driven learning schemes for identifying ablation targets (Lozoya et al., 2019). These findings have important consequences for clinical decision-making as they indicate how mechanistic and statistical models work together to determine ablation targets (Ali et al., 2019; Muffoletto et al., 2019; Cámara-Vázquez et al., 2021; Gander et al., 2022).

Moreover, a digital twin heart may indicate a CA strategy is appropriate for a patient by predicting the likelihood of AF recurrence before a specific therapy is selected (Muffoletto et al., 2019; Shade et al., 2020; Seno et al., 2021; Roney et al., 2022). For example, in the study of Roney et al., AF patient-specific models incorporating fibrotic remodeling from LGE-MRI scans were constructed to test six different ablation approaches. A random forest classifier was subsequently trained to predict ablation response. The study found the surface areas of pre-ablation driver regions and of fibrotic tissue not isolated by the proposed ablation strategy are both important for predicting ablation outcome (Roney et al., 2020). In addition, Azzolin et al. developed a technology to tailor ablations in AF patient-specific models aiming to identify the most successful ablation strategy. They used the Pacing at the End of the Effective Refractory Period (PEERP) protocol to localize emergent AF episodes, and then connected localized ablations to the closest non-conductive barrier to prevent recurrence of AF (Azzolin et al., 2021b). This study found that the proposed Personalized Ablation Lines (PersonAL) plan, consisting of iteratively targeting emergent high dominant frequency regions, outperformed state-of-the-art anatomical and substrate ablation strategies (Azzolin et al., 2022b).

## 4 Challenges and perspectives for the digital twin heart in AF

Before considering the digital twin techniques to improve the clinical treatment strategy, it may be beneficial to assess the sources of current therapies. Currently, most drugs used for the treatment of AF, such as quinidine, flecainide, propafenone, amiodarone, dofetilide, sotalol, and dronedarone, are not developed specifically to target AF (Nattel et al., 2021b). This fact is related to the importance of ventricular tachyarrhythmia as a potentially fatal clinical target. However, as the importance of AF to public health becomes apparent, drug development targeting AF is booming. In the major interventional approaches, the surgical maze procedure is the first mechanism-targeted approach to AF pathophysiology, whereas the empirical PVI is the most effective catheter-based procedure (Noheria et al., 2008). However, the apparent failure of AF treatment has primarity been attributed to the limited efficacy of AADs and the suboptimal PVI.

**FIGURE 2:**
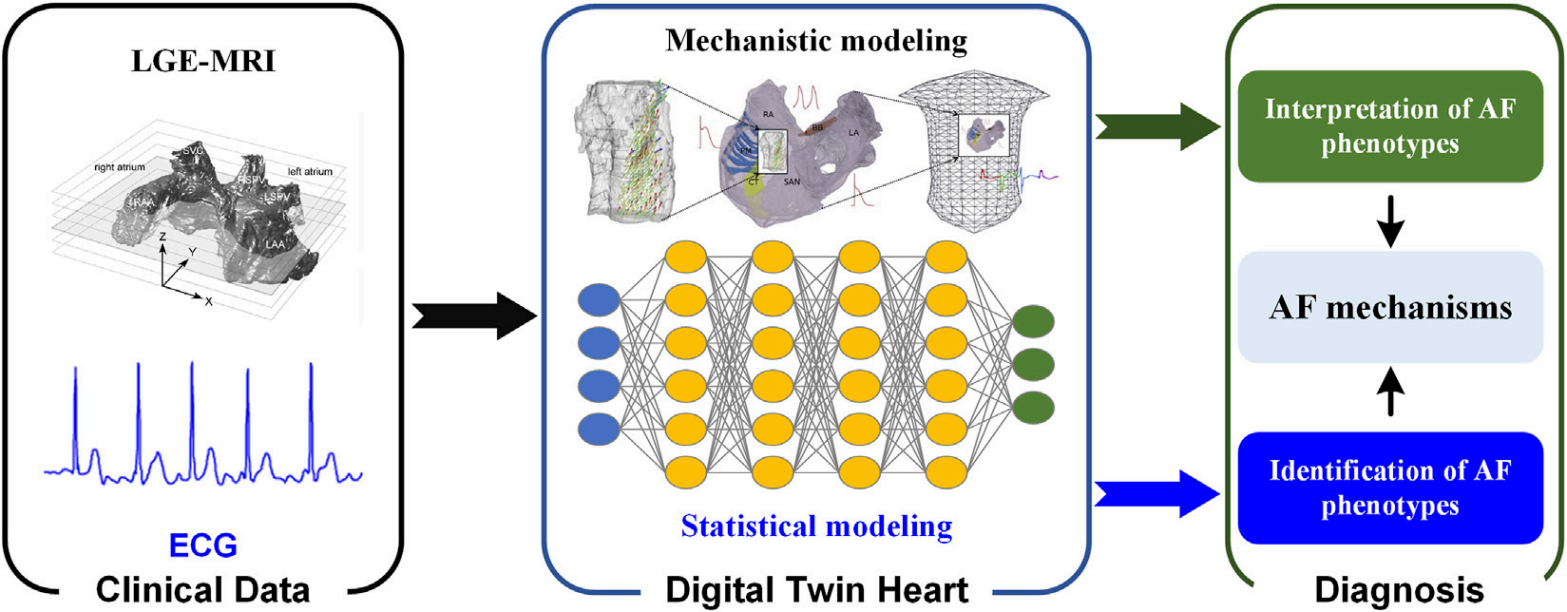
Digital twin heart in exploring the AF mechanisms. Clinical data are used to create and validate statistical and mechanistic models. Synergy between mechanistic and statistical models gives valuable insight that is clinically interpreted and combined with traditional data to aid in the process of clinical decision-making.

A digital twin heart that promises to transform from AF description to response prediction (i.e., from understanding AF mechanisms to screening anti-AF drugs and optimizing AF ablation strategies). In the digital twin heart, on the one hand, potential pathological mechanisms are explored through personalized multi-scale modeling and simulation; on the other hand, AF phenotypes are identified through a data-driven statistical model. Mechanistic and statistical models complement each other’s strengths to facilitate AF mechanism understanding and therapeutic evaluation. As experimental methods and imaging techniques continue to advance, more abundant and high-quality data will facilitate the development of digital twin hearts. Standardization of data acquisition and improved attention to re-usability will accelerate the development of digital twin technologies (Strocchi et al., 2020), while their integration into existing workflows will facilitate its clinical application. In the future, AF patients can be screened based on ECG biomarkers using statistical models (Xiong et al., 2018), while personalized biophysically detailed computational models of the atria based on the patient’s LGE-MRI can be used to interpret AF phenotypes (Figure 2) (Aslanidi et al., 2011).

Although there is a palpable exuberance in AF research regarding the potential of digital twin techniques, limitations of the various approaches and challenges in ensuring their clinical application remain. Whether it is the development of digital twin hearts or their clinical applications, the main challenge is the limited availability of experimental data at present. In order to achieve tailored AF treatment, we need to develop a more detailed personalized mechanistic model, but the functional and structural data required to build personalized atria are lacking. Except for the electrophysiological function data of the right atrial appendage of AF patients, other microstructural data, especially from the healthy atrium, are currently very scarce. Although individual structural data represented by patient-specific anatomy and fibrosis distribution can be obtained with LGE-MRI, the limited spatial resolution makes modeling fiber orientations and atrial fibrosis patterns difficult. Even if patient-specific models of the heart can be personalized, we still need to address the issue of intra-individual heterogeneity, including variability in atrial structural and functional properties. Due to the lack of massive experimental and clinical data, these heterogeneous features and their effect on the overall behavior of AF are poorly understood. For statistical models, supervised algorithms require significant amounts of high-quality labeled data. Annotation of data with labels is labor-intensive and datasets with poor data seriously affect the performance of algorithms. Therefore, data with its many aspects presents challenges to the digital twin heart adoption in AF management.

## 5 Conclusion

AF continues to pose a significant risk of death, in part due to knowledge gaps in the fundamental AF mechanisms and treatment strategies. These clinical challenges in understanding AF mechanisms, screening anti-AF drugs and optimizing AF ablation strategies might benefit from the digital twin techniques. Although limited by the availability of experimental data, the digital twin heart remains a promising path towards the vision of precision cardiology and its clinical applications are emerging.
